# The Histone Deacetylases HosA and HdaA Affect the Phenotype and Transcriptomic and Metabolic Profiles of *Aspergillus niger*

**DOI:** 10.3390/toxins11090520

**Published:** 2019-09-07

**Authors:** Xuejie Li, Lijie Pan, Bin Wang, Li Pan

**Affiliations:** School of Biology and Biological Engineering, South China University of Technology, No. 382 Waihuan East Rd, Guangzhou Higher Education Mega Center, Guangzhou 510006, China; bixuejieli@mail.scut.edu.cn (X.L.); bilijiepan@mail.scut.edu.cn (L.P.)

**Keywords:** *Aspergillus niger*, histone deacetylases, stress response, drug resistance, transcriptome, secondary metabolism

## Abstract

Histone acetylation is an important modification for the regulation of chromatin accessibility and is controlled by two kinds of histone-modifying enzymes: histone acetyltransferases (HATs) and histone deacetylases (HDACs). In filamentous fungi, there is increasing evidence that HATs and HDACs are critical factors related to mycelial growth, stress response, pathogenicity and production of secondary metabolites (SMs). In this study, seven *A. niger* histone deacetylase-deficient strains were constructed to investigate their effects on the strain growth phenotype as well as the transcriptomic and metabolic profiles of secondary metabolic pathways. Phenotypic analysis showed that deletion of *hosA* in *A. niger* FGSC A1279 leads to a significant reduction in growth, pigment production, sporulation and stress resistance, and deletion of *hdaA* leads to an increase in pigment production in liquid CD medium. According to the metabolomic analysis, the production of the well-known secondary metabolite fumonisin was reduced in both the *hosA* and *hdaA* mutants, and the production of kojic acid was reduced in the *hdaA* mutant and slightly increased in the *hosA* mutant. Results suggested that the histone deacetylases HosA and HdaA play a role in development and SM biosynthesis in *A. niger* FGSC A1279. Histone deacetylases offer new strategies for regulation of SM synthesis.

## 1. Introduction

Transcriptional regulation in filamentous fungi is strongly influenced by histone posttranslational modifications such as methylation, acetylation, phosphorylation, ubiquitylation and sumoylation [1,2]. The acetylation of N-terminal core histone tails is of utmost importance, and this dynamic process of acetylation is controlled by two histone modification enzymes with opposing activities: histone acetyltransferases (HATs), which transfer acetyl groups from acetyl-CoA to the ε-amino groups of lysine residues and histone deacetylases (HDACs), which catalyze the removal of this modification [1]. Histone acetylation is generally associated with elevated transcription, while deacetylated histones are often associated with gene repression [2]. However, there is still evidence that the deacetylation of histones could contribute to transcriptional activation as well [3].

In recent decades, histone acetylation/deacetylation has attracted extensive attention because this process was found to significantly affect the production of secondary metabolites (SMs). In *Aspergillus oryzae*, the deletion of *hdaB*, *hdaD*, and *hdaA* led to a significant decrease in the amount of N-acetylglucosamine in rice-koji [4]. Furthermore, the fungus-specific HstD/AoHst4 coordinates fungal development and secondary metabolism via the regulation of LaeA in *A. oryzae* [5]. The *ΔhdaA* mutant in *A. nidulans* exhibited increased production of metabolites (sterigmatocystin and penicillin) but unaltered terraquinone A (TR) levels [6]. In *Aspergillus fumigatus*, the HdaA is involved in the regulation of secondary metabolite production, and the deletion of the *hdaA* gene increased the production of several secondary metabolites while decreasing the production of gliotoxin, whereas overexpression of HdaA increased the production of gliotoxin [7]. In *Magnaporthe oryzae*, the deletion of *ΔMohda1*, an ortholog of yeast *hda1*, led to a significant stimulation of dark pigmentation in liquid culture. Metabolite analysis of a *ΔMohda1* mutant culture revealed that the accumulation of shunt products of the 1,8-dihydroxynaphthalene melanin and ergosterol pathways were significantly enhanced in the mutant strain compared to the wild-type strain [8,9]. The deletion of *hdf1* in *Fusarium graminearum* resulted in a significant reduction in virulence and deoxynivalenol (DON) production [10].

*Aspergillus niger* is used for the production of citric acid, extracellular enzymes, and heterologous proteins [11]. *A. niger* is also an important producer of SMs including polyketides, non-ribosomal peptides, indole alkaloid and terpenes [12]. Bioinformatics analysis indicates that *A. niger* encodes about 81 putative SM biosynthetic gene clusters. However, most of these putative gene clusters are silent in standard laboratory cultivation conditions. *A. niger* FGSC A1279, derived from NRRL3 (ATCC 9029, used for gluconic acid production) [13], is an SM-silent strain [14]. The low background of SM production in FGSC A1279 makes it ideal for investigating the role of epigenetic regulators on SM production.

In this study, to understand the role of HDACs in the regulation of SM biosynthesis in *A. niger* FGSC A1279, we constructed mutant strains for seven HDAC genes annotated in the ASPGD database. We revealed the effects of seven HDACs on the environmental stress and drug resistance by examining the phenotypic changes. Additionally, based on comparative transcriptomics between wild-type strains, *hosA* mutant (*ΔhosA*) and *hdaA* mutant (*ΔhdaA*), as well as chemical analysis, we aimed to survey the effects of HosA and HdaA on SM profiles. Since HosA and HdaA may regulate SM gene clusters, these results could provide novel potential resources for new SMs exploration.

## 2. Results

### 2.1. Phylogenetic Analysis Predicted the Amino Acid Sequences of HDACs in A. niger FGSC A1279 and Related Genes

**Figure 1:**
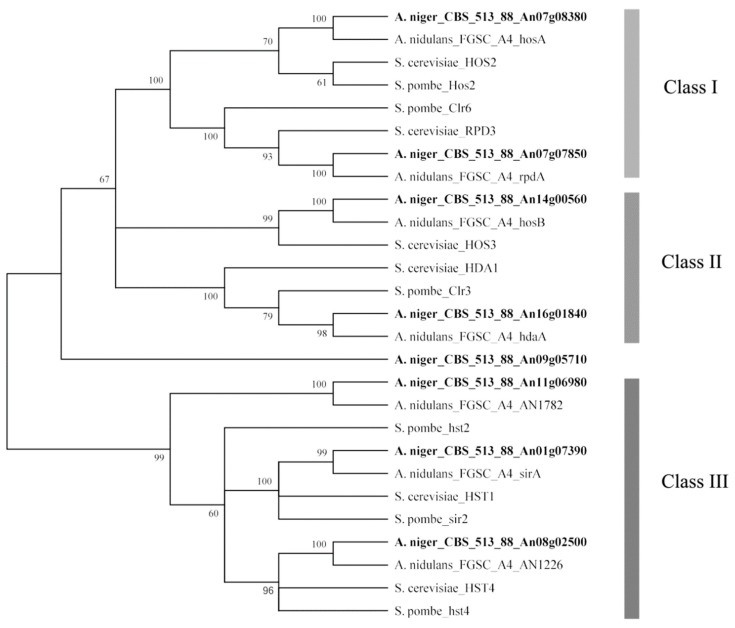
Neighbor-joining tree of histone deacetylases from *A. niger*, *A. nidulans*, *S. cerevisiae*, and *S. pombe*. The tree was constructed with the MEGA6 software by multiple amino acid sequence alignment. Bootstrap values are indicated on the node of each branch. The classes of HDACs are shown on the right. These classes of HDACs are described in a previous phylogenetic study [16,18]. HDACs of *A. niger* FGSC A1279 are indicated in bold.

A total of eight HDACs were predicted in the *A. niger* genome based on the genome annotation in the ASPGD database (Table 1). To classify these eight HDACs, all of these HDAC amino acid sequences (Figure S1) were subjected to phylogenetic analysis with known HDAC amino acid sequences from three different species: *S. cerevisiae*, *S. pombe*, and *A. nidulans*. An unrooted phylogenetic tree was generated based on amino acid sequence alignment using the MEGA6 program (Figure 1). These HDACs were phylogenetically divided into class I to III HDACs according to a previous phylogenetic study of HDACs [15,16,17], class I contributing to the major part of total HDAC activity, class II contributing to a minor part. Both class I and class II HDACs (except for HosB) were sensitive to trichostatin A and other hydroxamate analogs and class III HDACs required nicotinamide adenine dinucleotide (NAD^+^) for their activity. Figure 1 displays the predicted HDACs of *A. niger* that showed strong similarity to the HDACs of the other three species. Two HDAC genes, predicted to encode class I enzymes, were identified: *hosA* (An07g08380) and *rpdA* (An07g07850); two HDAC genes were classified as class II, including *hdaA* (An16g01840) and *hosB* (An14g00560); three genes, *hst2* (An11g06980), *hst4* (An08g02500) and *sirA* (An01g07390), belonged to class III, which were also described as sirtuin-type HDACs; another gene (An09g05710) with low homology to HDAC genes of the other three divergent species is also listed in Table 1.

### 2.2. HDAC-Deficient Strain Construction and Phenotype Analysis

**Figure 2:**
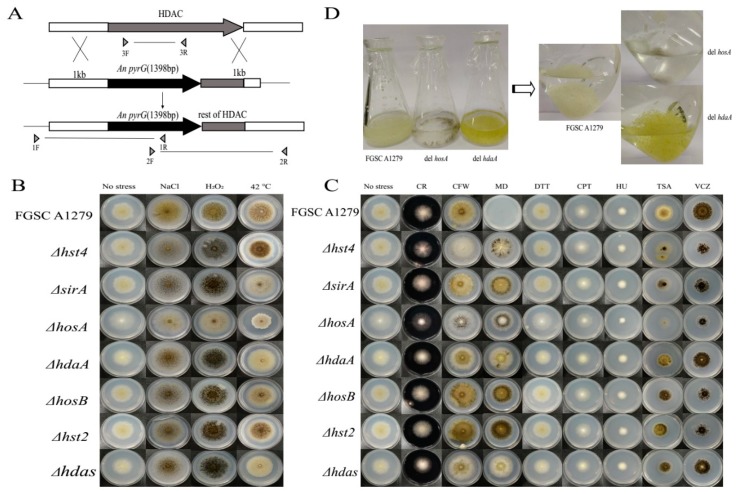
Disruption of HDAC genes in *A. niger* FGSC A1279 and phenotypic plates. (**A**) Each HDAC coding sequence was replaced with the pyrG selection marker by double homologous recombination. Positive deletion strains were confirmed by PCR screening with three different primer pairs. (**B**) Morphological phenotypes of disruptants on a stress culture plate on day seven. (**C**) Morphological phenotypes of the indicated strain in various chemical substances treated cultures after five days of growth. (**D**) Wild-type strains and the *ΔhosA*, and *ΔhdaA* mutants in liquid CD medium on day four. Abbreviations of chemical substances are listed in Table 2.

To investigate the association of each HDAC gene with the morphology and physiology of *A. niger* FGSC A1279, HDAC-deficient strains were constructed by homologous recombination (Figure 2A and Figure S2). Mutants were successfully obtained for seven HDACs. However, for *rpdA*, only heterokaryon transformants were obtained over several trials. This result suggested that *rpdA* is likely to be an essential gene in *A. niger* FGSC A1279, similar to the function of *rpdA* in *A. oryzae* or *A. nidulans* [5,19].

It has been reported that HDACs significantly influence stress tolerance [4,18,20] and virulence-associated phenotypes [21,22]. To decipher the possible physiological changes caused by the loss of the HDAC genes *in A. niger* FGSC A1279, HDAC-deficient mutants, and the corresponding controls, as listed in Table 1, were grown on media with various cultivation conditions. The sensitivity against osmotic (0.8 M NaCl), oxidative (10 mM H_2_O_2_) and heat stress (42 °C) was evaluated for the following parameters: extension and branching of the mycelia, conidiation and colony diameter (Figure 2B). The *ΔhosA* strain showed significant phenotypic changes under heat, oxidative and osmotic stress. Specifically, the spore count decreased considerably under oxidative and osmotic stress, and the colony diameter of the *ΔhosA* strain decreased considerably at 42 °C (Figure 2B and Figure S3A). The *ΔhdaA*, *ΔsirA* and *Δhdas* strains were sensitive to heat, and the formation of conidia and melanin by these three disruptants decreased (Figure 2B and Figure S3B).

Then, we examined the resistance of HDAC-deficient mutants to various chemicals, including CR, CFW, MD, DTT, CPT, HU, TSA, and VCZ (Table 2). The *ΔhosA* strain showed the greatest sensitivity to CR, CFW, DTT, TSA, and VCZ. The *ΔhdaA* strain was slightly sensitive to CFW and DTT. The *Δhst4* disruptant was sensitive to CFW and TSA. The *Δhdas* strain looks less green when media supplemented with CFW or DTT. All seven HDAC disruptants were resistant to MD. The *Δhst2*, *ΔsirA*, and *ΔhosB* disruptants had no significant phenotypic differences compared with the controls under different culture conditions (Figure 2C). We speculated that these three HDACs have little impact on phenotype in *A. niger* FGSC A1279. These findings suggest that the deletion of *hosA* significantly affects the morphology of *A. niger* FGSC A1279, and *hosA* is required for stress tolerance and drug resistance.

It has been reported that HdaA contributes to a majority of the HDAC activity and regulates the production of secondary metabolites in *A. nidulans* [6,20], *A. fumigatus* [7], and *A. oryzae* [4,5]. In the follow-up study, we mainly focused on the genes *hosA* and *hdaA*. We additionally observed the growth of the *ΔhosA* and *ΔhdaA* mutants and wild-type strains in liquid CD medium. Figure 2D shows that compared with the wild-type strains, *ΔhdaA* mutant have exhibit significant pigment accumulation and *ΔhosA* mutant show growth retardation. These results demonstrated that the deletion of *hdaA* activated pigment synthesis in liquid CD medium cultivation, while the deletion of *hosA* decreased resistance to stress and some chemicals.

### 2.3. Phenotype Analysis of HosA and HdaA Complementation Strains

**Figure 3:**
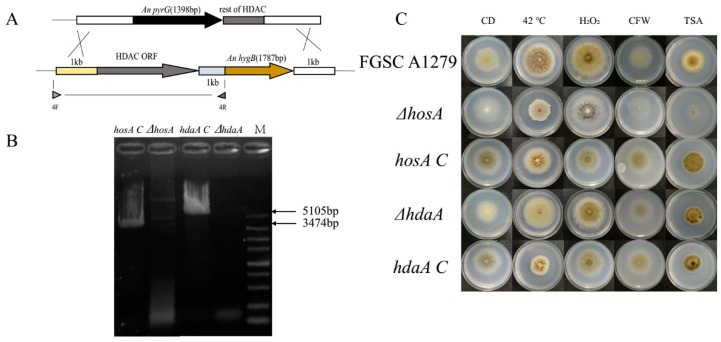
Construction of *hosA* and *hdaA* complementation strains and phenotypic plates. (**A**) *hosA* and *hdaA* were complemented with a hygromycin selection marker and the HDACs with their own promoters and terminators. (**B**) Agarose gel electrophoresis of the PCRs that were used to confirm that the *hosA* and *hdaA* expression cassette were integrated into the genome. Lane M, DL5000 bp DNA Marker (Takara); lane 1, PCR-amplified DNA fragments using the primer pair of *hosA* shows as 4F and 4R; lane 3, PCR-amplified DNA fragments using the primer pair of *hdaA* shows as 4F and 4R; lane 2 and lane 4 were control groups. The template of each PCR is marked on each lane. (**C**) Morphological phenotypes of wild-type strains, the *ΔhosA* and *ΔhdaA* mutants and the complementation strains under culture conditions in which the phenotypes of the two mutants varied significantly.

To further confirm the role of *hosA* and *hdaA* on phenotypic changes, complementation strains of these two genes were constructed (Figure 3A,B). The genes *hosA* and *hdaA*, with their own promoters and terminators, were reintegrated into chromosomal sequences. The strains, including *ΔhosA*, *ΔhdaA*, *hosA C* (*hosA* complementation stain)*, hdaA C* (*hosA* complementation stain) and *A. niger* FGSC A1279, were cultivated on CD medium supplemented with 10 mM H_2_O_2_, 300 μg/mL CFW, or 5 μg/mL TSA at 30 °C or 42 °C (Figure 3C). The *ΔhosA* strains showed significant defects in response to heat, 10 mM H_2_O_2_, 300 μg/mL CFW, and 5 μg/mL TSA. As expected, the phenotype of *the hosA C* strain was similar to that of *A. niger* FGSC A1279. The *ΔhdaA* strain showed a slight sensitivity to 10 mM H_2_O_2_ and heat. The *hdaA C* strain produced more pigment and spores at 30 °C in CD medium than the *ΔhdaA* strain (Figure 3C and Figure S3C). The colonies of the *hdaA C* strain were plicated, similar to the phenotype of the wild-type strains at 42 °C, while the colony diameter of *hdaA C* strains was shorter than wild-type strains (Figure S3D). The phenotypes of the *hosA C* and *hdaA C* strains basically reverted to the wild-type phenotype, suggesting that the *hosA* and *hdaA* genes had been successfully complemented. We hypothesized that *hosA* and *hdaA* play an important role in stress tolerance and drug resistance.

### 2.4. Transcriptomic Profiles of A. niger FGSC A1279, ΔHosA and ΔHdaA

To investigate the effects of HosA and HdaA on the transcriptome of *A. niger* FGSC A1279, we compared gene expression in *A. niger* FGSC A1279 and the *hosA* and *hdaA* mutants using high-throughput RNA-Seq with a strand-specific paired-end sequencing strategy. Two biological replicates were analyzed, and a total of 23.09 (88.17%), 23.08 (88.50%), and 22.95 (87.69%) million reads were obtained, and numbers listed in brackets means the percent of reads uniquely mapped to the *A. niger* reference genome (Table S1).

Alignments were analyzed with DEGseq [23] to conduct a genome-wide analysis of differential gene expression. Compared to the WT strain, the expression of 656 genes in the *ΔhdaA* strain was significantly upregulated (fold change ≥ 2, FDR ≤ 0.001), while the expression of 394 genes was downregulated. Compared to the WT strain, the expression of 433 genes in the *Δhos2* strain was significantly upregulated (fold change ≥ 2, FDR ≤ 0.001), while the expression of 1967 genes was downregulated (Supplementary Figure S4).

**Figure 4:**
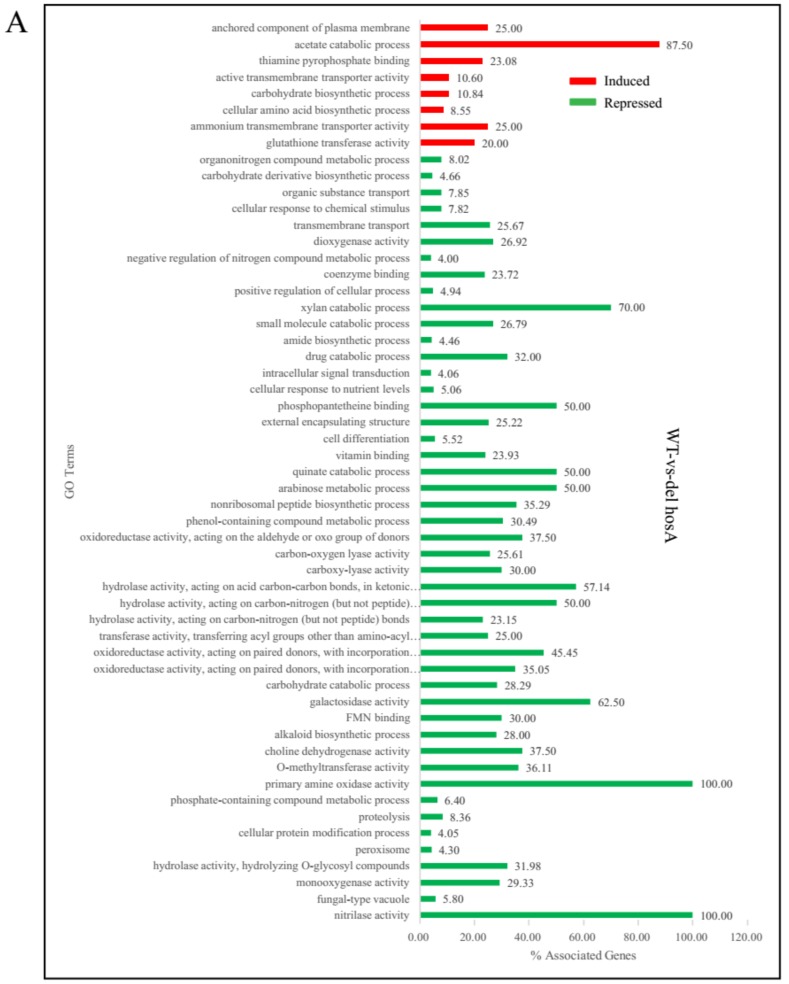

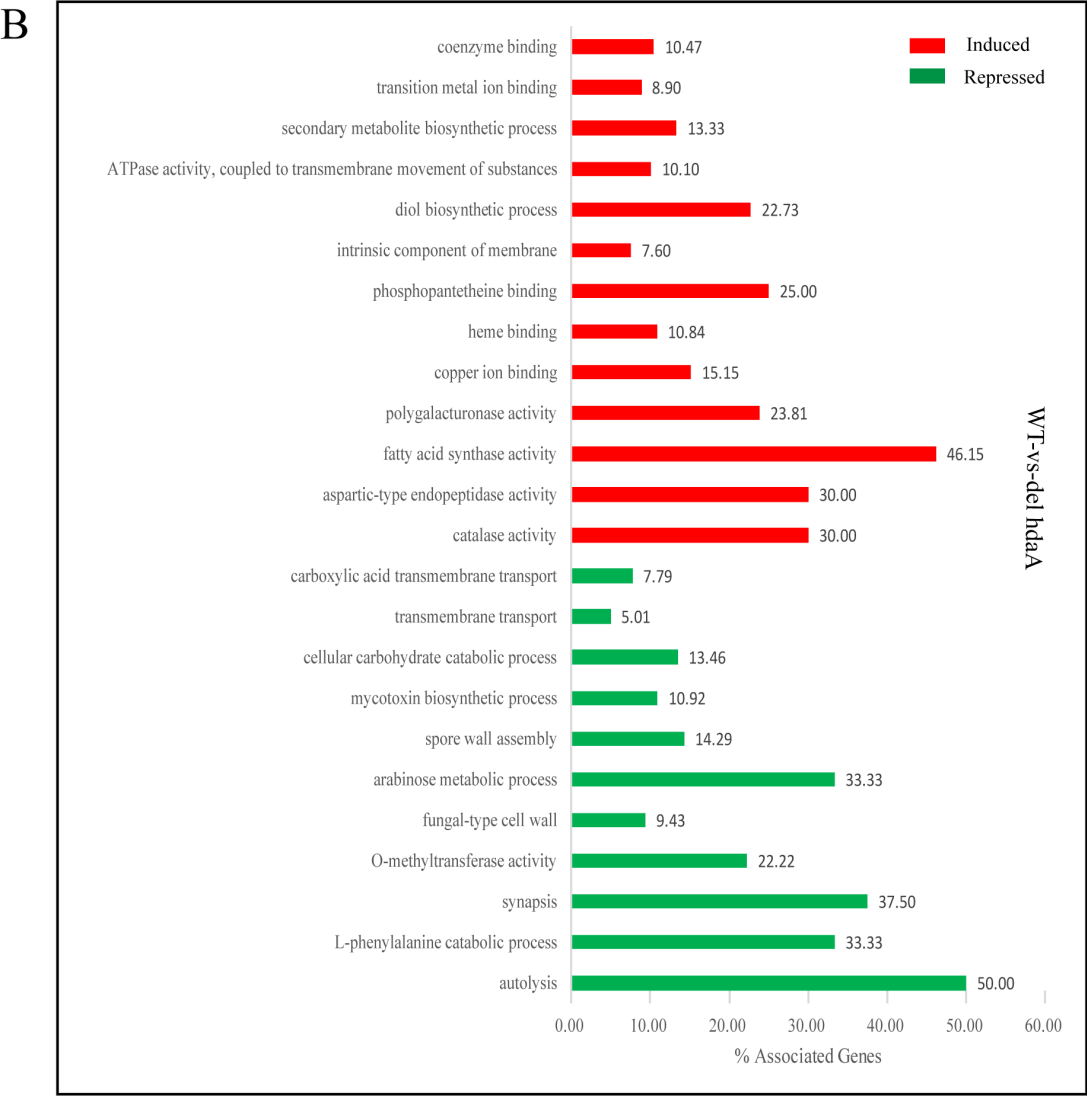
GO enrichment analysis of differentially induced or repressed genes in the *ΔhosA* and *ΔhdaA* mutants. (**A**) Representation of the main significantly induced and repressed pathways (*p* < 0.001) in the *ΔhosA* mutant strain compared with the wild-type strains. (**B**) Representation of the main significantly induced and repressed pathways (*p* < 0.001) in the *ΔhdaA* mutant strain compared with the wild-type strains.

To reveal the functional distribution of genes affected by HosA and HdaA, ClueGo enrichment analysis was carried out using the *A. niger* FGSC A1279 strain as control via Cytoscape software. For *ΔhosA* strains, the upregulated genes were enriched in eight GO terms. The downregulated genes were enriched in 47 GO terms, including carbohydrate (xylan, arabinose, galactosidase) catabolic process, secondary metabolic process (nonribosomal peptide and alkaloid) (Figure 4A). According to RNA-Seq analyses, we speculated that the main reason that the hyphal growth of *ΔhosA* mutant became slow on CD medium is that the expression of genes related to carbohydrate metabolic process was repressed in *ΔhosA* mutant. The expression of secondary metabolites in *ΔhosA* mutant was also inhibited.

For the *ΔhdaA* strain, the upregulated genes were enriched in 13 GO terms, including partial secondary metabolite biosynthetic process. Additionally, the downregulated genes were enriched in 11 GO terms, including spore wall assembly, fungal-type cell wall, mycotoxin biosynthetic process, and carbohydrate (arabinose) catabolic process (Figure 4B). We assumed that HdaA affected the formation of spores and cell walls and the production of secondary metabolites.

### 2.5. Effect of HosA and HdaA on Asexual or Sexual Reproduction, Pigmentation, and Cell Wall Synthesis

**Figure 5:**
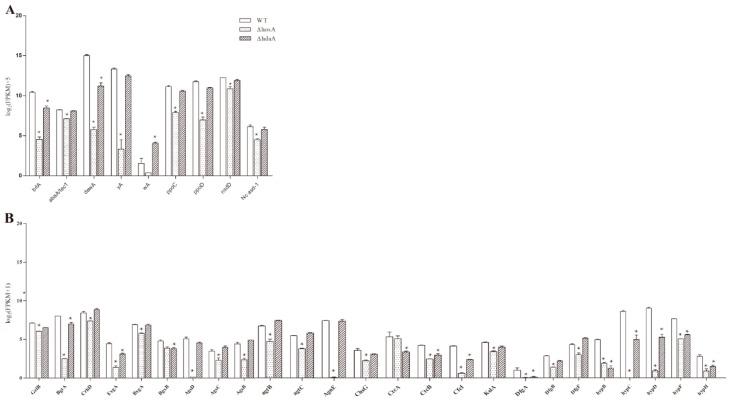
Analysis of differentially expressed genes related to asexual or sexual reproduction and cell wall biosynthesis in the wild-type strains and the *ΔhosA* and *ΔhdaA* mutants. (**A**) Bar graphs show the expression values (log_2_ [FPKM]+5) for annotated proteins associated with asexual or sexual reproduction differentially expressed in the wild-type strains and the *ΔhosA* and *ΔhdaA* mutants. (**B**) Bar graphs show the expression values (log_2_ [FPKM+1]) for annotated proteins associated with cell wall biosynthesis in the wild-type strains and the *ΔhosA* and *ΔhdaA* mutants. * represents significant changes.

In the *ΔhosA* strain, the significantly downregulated (fold change ≥ 2, FDR ≤ 0.001) genes related to asexual or sexual reproduction were *brlA*, *abaA*, *dewA*, *yA*, *ppoC*, *ppoD*, *nsdD*, and *Nc asd-1* (Figure 5A and Table S2). The decreased expression of *yA*, responsible for pigment synthesis, led to little pigment being produced in *the ΔhosA* strain. The expression of *brlA* and *dewA* was downregulated in the *ΔhdaA* strain, and the expression of *wA*, related to pigment biosynthesis, was upregulated. These results are consistent with the result that the *ΔhosA* strain produced fewer spores than the wildtype strain.

Both *hosA* and *hdaA* affected cell wall synthesis (Figure 5B and Table S3). In the *ΔhosA* and *ΔhdaA* strains, the expression of five out of eight genes related to hydrophobin synthesis [24], namely, *hypB*, *hypC*, *hypD*, *hypF*, and *hypH*, was significantly downregulated. Moreover, the expression of 18 other genes related to cell wall synthesis was also downregulated in *ΔhosA* strains. In the *ΔhdaA* strain, the expression of eight genes related to cell wall synthesis was downregulated (Figure 5B). These results demonstrate that the deletion of *hosA* significantly affected the production of conidia, pigments and cell wall. Deletion of *hdaA* caused upregulation of the pigment associated gene *wA* and downregulation of genes related to conidia and cell wall. Compared with *hdaA*, the deletion of *hosA* significantly affected phenotypic changes.

### 2.6. Effects of the Genes HosA and HdaA on Secondary Metabolites

Because the *ΔhdaA* strain accumulated more pigment than wild-type strains in the liquid CD medium (Figure 2D), we were interested in whether the deletion of *hosA* and *hdaA* affects SM production in *A. niger* FGSC A1279. To address this question, RNA-Seq data for 70 SM backbone genes identified in the genome of *A. niger* FGSC A1279 were analyzed with regard to transcription (Supplementary Table S4). A heatmap of 70 backbone genes of SM clusters illustrates that compared with wild-type strain, backbone genes of SM clusters were mostly downregulated in the *ΔhosA* strain, while backbone genes of SM clusters were mostly upregulated in the *hdaA* mutant (Figure S5).

**Figure 6:**
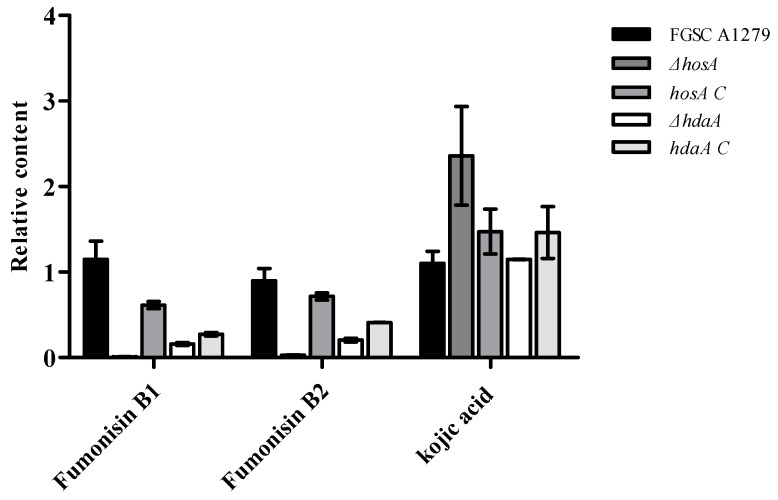
Analysis of the production levels of fumonisin B1, fumonisin B2, and kojic acid (KA) in the wild-type (WT) strains, *ΔhosA* mutant, *ΔhdaA* mutant, *hosA C*, and *hdaA C* strains. Bar graphs show the relative content of fumonisin B1, fumonisin B2, and KA in the strains after seven days of growth in WATM at 30 °C. The WT strain was used as a control. Data are analyzed using Compound Discoverer (*n* = 2).

To further confirm the effects of HosA and HdaA on secondary metabolites, the metabolites of the *ΔhosA* and *ΔhdaA* mutants and wild-type strains were analyzed. Consistent with genomics data, chemical analysis showed that fumonisin B1 and fumonisin B2 were present among the metabolites of *A. niger*, but no ochratoxin A was detected. For the *ΔhosA* mutant, the production of fumonisin B1 and B2 was reduced (fold change ≥ 2) compared with that for the wild-type strains (Supplementary Figure S6, Tables S5 and S6). The production of fumonisin B1 and B2 in the *hosA C* strains was higher than the production in the *ΔhosA* mutant and was slightly lower than the production in the wild-type strains (Figure 6 and Table S6). The production of fumonisin B1 and B2 in the *ΔhdaA* mutant significantly decreased compared with that in the wild-type strains (Figure S6, Tables S7 and S8). For the *hdaA C* strains, the production of fumonisin B1 was lower than the production in the wild-type strains and was approximately equal to the production in the *ΔhdaA* mutant. The production of fumonisin B2 in the *hdaA C* strains was higher than the production in the *ΔhdaA* mutant (Figure 6 and Table S8).

Then, we investigated the potential capacity of *A. niger* FGSC A1279 to secrete other secondary metabolites. Kojic acid (KA), which is widely used in cosmetics as a skin-lightening agent and is usually found in *Aspergillus oryzae* [25,26], has been detected in *A. niger* FGSC A1279. The production of KA was higher (fold change ≥ 1.5) in the *ΔhosA* mutant and did not significantly changed in the *ΔhdaA* mutant than in the wild-type strains (Figure 6). The production of KA in *hosA C* strains was lower than in the *ΔhosA* mutant and the production of KA in *hdaA C* strains was higher than in the *ΔhdaA* mutant.

## 3. Discussion

Chromatin modifications have important effects on transcriptional regulation [27]. Among histone modifications, acetylation and deacetylation are effective methods to regulate SM clusters and pathogenicity in fungi [6,28]. The genomic sequencing analysis, coupled with chemical analysis studies of *A. niger*, revealed that a large number of genes putatively involved in SM synthesis are silent in laboratory conditions [29]. To investigate the effect of HDACs on *A. niger*, eight HDAC genes encoding orthologs of histone deacetylases of *A. nidulans* or *S. cerevisiae* were phylogenetically analyzed and separately deleted in *A. niger* FGSC A1279. Except for the An09g05710 gene, the other seven genes showed high homology with HDACs in *A. nidulans*, *S. cerevisiae*, and *S. pombe.*

In contrast to the successful deletion of seven other HDACs, all efforts to obtain RPDA-type null mutant failed, suggesting that RpdA may be essential for the recovery of transformed protoplasts. As reported previously, RpdA is essential for the viability of *A. nidulans.* Conditional knockout of the *rpdA* gene shows that *rpdA* depletion significantly affects fungal growth and sporulation [19,30]. It is likely that RpdA in *A. oryzae* is also essential for survival and is involved in various stress responses [4,5,18]. In the corn smut fungus *Ustilago maydis*, a RpdA-related enzyme is necessary for the proliferation of diploid cells during teliospore development [31]. Deletion of *rpd3* in *Beauveria bassiana* results in severe growth defects, reduction in conidiation capacity and drastic attenuation in virulence [32].

The *hosA* deletion in FGSC A1279 had obvious vegetative growth effects on both solid and liquid CD medium. The *ΔhosA* mutant was highly sensitive to environmental stress and chemical drugs (Figure 2B,C). The phenotype of *hosA C* strains was similar to the phenotype of the wild-type strains. Phenotype assays suggested that in *A. niger* FGSC A1279, HosA played the most important role in the phenotype, including hyphal growth, pigment accumulation, and spore count of eight histone deacetylases. In *Beauveria bassiana*, deletion of *hos2* increased the sensitivity to oxidative stress and reduced conidiation capacity [33]. The synthesis of SMs and proteins involved in virulence and disease are significantly affected by the deletion of HosA [34]. Chemical analysis showed that in *A. niger* FGSC A1279, the production of fumonisin B1 and B2 was significantly reduced and that the production of KA increased. We assume that HosA and many other factors, including chromosome localization, together affect the production of SMs. In addition to being linked to histone deacetylation, HosA may be responsible for other epigenetic modifications. In *Beauveria bassiana*, the histone deacetylase Hos2 plays an indirect role in acetylating H3-K56 and phosphorylating H2A-S129 [33].

The loss of HdaA had an insignificant influence on mycelial growth, pigment accumulation and environmental pressure resistance compared with HosA (Figure 2B,C). Similarly, in *A. fumigatus*, the loss of *AfhdaA*, homologous gene *hdaA*, did not affect growth under oxidative stress7 conditions [7]. However, HdaA is involved in the regulation of enzymes which are of vital importance for the cellular antioxidant response in *A. nidulans*. *ΔhdaA* strains exhibit significantly reduced growth under oxidative stress conditions [20]. The possible reason the phenotype of *hdaA C* strains is slightly different from the phenotype of the wild-type strains is that the *hdaA C* expression cassette was not in situ integration in the genome. Transcriptomic analysis of the *ΔhdaA* mutant suggests that in *A. niger* FGSC A1279, HdaA activates the transcription of some of the SM backbone genes. However, for fumonisin B1 and, B2, the chemical analysis showed that these two kinds of metabolites were downregulated. In *A. nidulans*, the telomere-proximal gene clusters of mycotoxin, sterigmatocystin and antibiotic penicillin were specifically upregulated in the *hdaA* deletion strain [6]. In *A. fumigatus*, however, deletion of *hdaA* resulted in both positive and negative regulation of secondary metabolite genes [7]. In *Fusarium fujikuroi*, deletion of an *hdaA* ortholog not only altered the number of secondary metabolites such as gibberellic acids, fusaric acid, and bikaverin but also abrogated the regulation of bikaverin biosynthesis [35]. Taken together, these data suggest that HdaA can modulate the expression of secondary metabolite cluster genes, either positively or negatively, in filamentous fungi.

Deletion of the other five HDAC genes in *A. niger* FGSC A1279 had almost no effect on vegetative growth, reduction in conidiation or pigment production. However, it was shown that members of other HDACs have additional roles beyond the deacetylation of histones and mediate diverse biological functions. For example, in *A. nidulans* HosB has little effect on phenotypic changes but may be involved in regulating secondary metabolism [16,20]. Sirtuin class histone deacetylase, HstA, has been reported to be involved in the production of penicillin in *A. nidulans* [6]. Hst4 in *A.oryzae* is associated with the regulation of growth, stress responses, asexual development and transcription of secondary metabolism-related genes [4,5]. To verify the functions of the other five HDACs, additional experiments are needed, and these five HDAC genes may yet still prove to impact SM production.

In contrast to previous reports that HDACs are associated with the formation of heterochromatin, transcription inhibition, and gene silencing. Genome-wide transcriptome analysis in our study revealed that the expression of several enzymes associated with carbohydrate metabolic processes and the transcription of multiple genes involved in sexual or asexual reproduction and cell wall biosynthesis were significantly repressed in the *ΔhosA* mutant. In our previous experiments, the deletion of the epigenetic regulator gcnE, a histone acetyltransferase in the SAGA/ADA complex, activated the production of secondary metabolites in *A. niger* FGSC A1279 [12]. Therefore, our results reveal that histone acetylation is not necessarily linked to gene activation and histone deacetylation associated with gene repression, and further detailed study is needed to verify the relationship between histone epigenetic modification and gene expression.

## 4. Materials and Methods

### 4.1. Phylogenetic Analysis of HDACs

The protein sequences of identified histone deacetylases from *Saccharomyces. cerevisiae* (*S. cerevisiae*), *Saccharomyces. pombe* (*S. pombe*), and *Aspergillus. nidulans* (*A. nidulans*) were obtained from different fungal genome databases: *Saccharomyces* genome database (https://www.yeastgenome.org/), PomBase (https://www.pombase.org/), and AspGD (http://www.aspgd.org/), respectively. These sequences were compared with HDAC gene homologs in *A. niger* CBS513.88. Multiple sequence alignments and phylogenetic analyses were performed using the MEGA6 program.

### 4.2. Strains and Growth Conditions

All *Aspergillus niger* strains used in this study are listed in Table 1. Strains were grown on potato dextrose agar at 30 °C for spore harvest. For fungal transformations and DNA extraction, strains were grown on DPY liquid medium (2% (*w*/*v*) glucose, 1% (*w*/*v*) polypeptone, 0.5% (*w*/*v*) yeast extract, 0.5% (w/v) KH_2_PO_4_, 0.05% (*w*/*v*) MgSO_4_·7H_2_O, pH 5.5) and cultured in a shaker at 200 rpm at 30 °C. Czapek Dox (CD) medium (2% (*w*/*v*) glucose, 0.3% (*w*/*v*) NaNO_3_, 0.1% (*w*/*v*) K_2_HPO_4_, 0.2% KCl, 0.05% (*w*/*v*) MgSO_4_·7H_2_O, and 0.001% (*w*/*v*) FeSO_4_·7H_2_O) was used as the basic medium for transformant identification and all stress and drug resistance assays. Solid WATM medium (0.2% (*w*/*v*) yeast extract, 0.3% (*w*/*v*) polypeptone, 0.2% (*w*/*v*) glucose, 3% (*w*/*v*)sucrose, 0.5% (*w*/*v*) corn steep solids, 0.2% (*w*/*v*) NaNO_3_, 0.1% (*w*/*v*)K_2_HPO_4_·3H_2_O, 0.5% (*w*/*v*) MgSO_4_·7H_2_O, 0.02% (*w*/*v*) KCl, 0.001% (*w*/*v*) FeSO_4_·7H_2_O pH 7.0) was used for SM profile analysis. All media contained appropriate supplements to maintain auxotrophs.

### 4.3. Construction of A. niger Histone Deacetylase Mutants

The deletion of eight putative histone deacetylases was performed via homologous replacement in *A. niger* FGSC A1279 as described previously [14], using the pyrG gene as the selection marker. The deletion cassette consists of three fragments: the 1-kb upstream region of the HDAC coding sequence, the pyrG gene (1398 bp) and the 1-kb downstream region containing a partial coding region. The deletion cassette was transformed into *A. niger* according to a method described previously [12]. The transformants were identified by PCR amplification of chromosomal sequences (Figure 2A and Figure S2). For complementation of *hosA* and *hdaA*, the expression cassette of *hosA* or *hdaA* was transformed into the corresponding deficient strains using a hygromycin B selection marker. The complementation strains were identified using resistance screening and PCR amplification (Figure 3A,B).

### 4.4. Environmental Stress Resistance and Drug Resistance Assay

For the phenotypic assay, 1 μL of a conidial suspension (1 × 10^7^ conidia) of each strain was inoculated on the center of a plate and grown for 7 days at 30 °C. For the osmotic and oxidative stress resistance tests, 0.8 M NaCl and 10 mM H_2_O_2_ were added to solid CD medium. For the heat resistance test, the plate was incubated at 42 °C. For drug resistance analysis, conidial suspensions of each strain were inoculated on CD medium supplemented with different chemicals, including: 300 μg/mL calcofluor white (CFW), 1 mg/mL Cargo red (CR) (Meryer, Shanghai, China), 10 mM hydroxyurea (HU) (Aery, Jinan, China), 3 μM camptothecin (CPT) (Research Accelerators, Chengdu, China), 45 μM menadione (MD) (Macklin, Shanghai, China), 500 ng/mL voriconazole (VCZ) (Lunarsun, Beijing, China), 20 μM dithiothreitol (DTT) (Macklin, Shanghai, China), and 5 μg/mL trichostatin A (TSA) (Topscience, Shanghai, China). The detailed functions of these chemicals are listed in Table 2.

### 4.5. RNA Preparation and RNA-Seq Analysis

A total of 5 × 10^6^ spores/plate of FGSC A1279 and the *ΔhosA* and *ΔhdaA* mutant strains were distributed over a CD medium agar plate in duplicate, which was covered with a layer of cellophane and cultivated at 30 °C until the mid-logarithmic phase (48 h). After cultivation, mycelia were harvested and immediately ground to a fine powder in liquid nitrogen and then stored at −80 °C. Total RNA from each sample, with a 28 S:18 S ratio >1.5 and an absorbance OD_260_/OD_280_ ratio between 1.8 and 2.0, was extracted using RNAiso^TM^ Plus (TaKaRa, Ohtsu, Shiga, Japan) according to the manufacturer’s instructions [14]. Subsequently, cDNA libraries for each sample were constructed using TruSeq RNA Sample Preparation Kits v2 (Illumina, San Diago, CA, USA) according to the manufacturer’s instructions. After quantification and qualification during the quality control step, the sample library was sequenced on the BGISEQ-500 platform. The raw sequencing data were deposited in the Sequence Read Archive (http://www.ncbi.nlm.nih.gov/sra/) at the National Center for Biotechnology Information under the accession number PRJNA554537. RNA-Seq data analysis was performed as described in the literature [36]. The heatmap was plotted using OmicShare Tools, a free online platform for data analysis (http://www.omicshare.com/tools).

### 4.6. Secondary Metabolite Collection and Analysis

To collect secondary metabolites, FGSC A1279 and its mutants (*hosA* and *hdaA*) were cultivated for 7 days at 30 °C in the dark on solid WATM medium. Cultures were extracted with an equal volume of ethyl acetate (EtOAc) plus 1% formic acid for 24 h. The crude extract was filtered and dried on a rotary evaporator at 38 °C, redissolved in 5 mL of MeOH and evaporated with pressure blowing concentrator. The dried metabolite pellets were redissolved in 80% methanol and analyzed by LC-MS/MS. LC-MS/MS analyses were performed using a Vanquish UHPLC system (Thermo Fisher Scientific, Waltham, MA, USA) coupled with an Orbitrap Q Exactive HF-X mass spectrometer (Thermo Fisher Scientific, Waltham, MA, USA) operating in the data-dependent acquisition (DDA) mode. Samples were injected onto an Hyperil Gold column (100 × 2.1 mm, 1.9 µm) using a 16-min linear gradient at a flow rate of 0.3 mL/min. The eluents of the positive polarity mode were eluent A (0.1% FA in water) and eluent B (Methanol). The eluents of the negative polarity mode were eluent A (5 mM ammonium acetate, pH 9.0) and eluent B (Methanol). The solvent gradient was set as follows: 2% B, 1.5 min; 2–100% B, 12.0 min; 100% B, 14.0 min; 100–2% B, 14.1 min; 2% B, 16 min. The Q-Exactive HF-X mass spectrometer was operated in positive/negative polarity mode with a spray voltage of 3.2 kV, capillary temperature of 320 °C, sheath gas flow rate of 35 arb and aux gas flow rate of 10 arb. The data were analyzed using the Compound Discoverer software.

## Figures and Tables

**Table 1:** Strains used in this study.

Name *	Genotype	Disrupted Gene ID	HDAC Type (HDAC Class)
FGSC A1279	*Δku70::ptrA ΔpyrG:amds*		
*ΔhosA*	*Δku70::ptrA ΔpyrG:amds ΔhosA::pyrG*	An07g08380	Classical HDAC (Class I)
*ΔrpdA ht* **	*Δku70::ptrA ΔpyrG:amds ΔrpdA::pyrG*	An07g07850	Classical HDAC (Class I)
*ΔhdaA*	*Δku70::ptrA ΔpyrG:amds ΔhdaA::pyrG*	An16g01840	Classical HDAC (Class II)
*ΔhosB*	*Δku70::ptrA ΔpyrG:amds ΔhosB::pyrG*	An14g00560	Classical HDAC (Class II)
*Δhst2*	*Δku70::ptrA ΔpyrG:amds Δhst2::pyrG*	An11g06980	sirtuins (Class III)
*Δhst4*	*Δku70::ptrA ΔpyrG:amds Δhst4::pyrG*	An08g02500	sirtuins (Class III)
*ΔsirA*	*Δku70::ptrA ΔpyrG:amds ΔsirA::pyrG*	An01g07390	sirtuins (Class III)
*Δhdas*	*Δku70::ptrA ΔpyrG:amds Δhdas::pyrG*	An09g05710	

* HDAC homolog of *Saccharomyces cerevisiae*, *Saccharomyces pombe* or *Aspergillus nidulans*. ** ht: heterokaryon.

**Table 2:** Chemical materials used in this study.

Name	Abbreviation	Concentration	Effects to Cellular Process
cargo red	CR	1 mg/mL	cell walls stress
calcofluor white	CFW	3 μg/mL	cell walls stress
menadione	MD	45 μM	oxidative
dithiothreitol	DTT	20 μM	reductive
camptothecin	CPT	3 μM	obstruct DNA replication and transcription
hydroxy urea	HU	10 M	obstruct DNA replication
trichostatin A	TSA	5 μg/mL	histone inhibitor
voriconazole	VCZ	20 ng/mL	cellular membrane stress

## Supplementary Materials

The following are available online at https://www.mdpi.com/2072-6651/11/9/520/s1, Figure S1: Amino acid sequences of eight HDACs in *A. niger* FGSC A1279 obtained from ASPGD, Figure S2: Disruption of HDAC genes in *A. niger* FGSC A1279, Figure S3: Statistical analysis of spore count and colony diameter, Figure S4: Overview of the number of differentially expressed genes via pairwise comparisons of the *ΔhosA* mutant and wild strains, *ΔhdaA* mutant and wild strains, Figure S5: Heatmaps display expression of backbone genes of SMs biosynthesis gene clusters in wild strains, *ΔhosA* and *ΔhdaA* mutants after seven days of growth on WATM at 30 °C, Figure S6: Analysis of the expression levels of kojic acid (KA), fumonisin B1 and fumonisin B2 in the wild-type strains and the ΔhosA and ΔhdaA mutants, Table S1: Summary of sequencing and reads mapping, Table S2: Expression level of genes related to asexual or sexual reproduction, Table S3: Expression level of genes related to cell wall biosynthesis, Table S4: Expression level of backbone genes in SMs gene clusters, Table S5: Data of metabolomic studies. Expression of kojic acid, fumonisin B1 and fumonisin B2 in wild strains and *ΔhosA* mutant, Table S6: Data of LC-MS. Relative output of kojic acid, fumonisin B1 and fumonisin B2 in *ΔhosA* mutant/WT and *hosA C*/WT, Table S7: Data of metabolomic studies. Expression of kojic acid, fumonisin B1 and fumonisin B2 in wild strains and *ΔhdaA* mutant, Table S8: Data of LC-MS. Expression of kojic acid, fumonisin B1 and fumonisin B2 in in *ΔhdaA* mutant/WT and *hdaA C*/WT.
