# Supplementary material for: The Histone Deacetylases HosA and HdaA Affect the Phenotype and Transcriptomic and Metabolic Profiles of Aspergillus niger
Source: Toxins (Basel). 2019 Sep 7;11(9):520. doi: 10.3390/toxins11090520 (PMC6784283; doi:10.3390/toxins11090520)
Supplement: Supplementary file 1 [file toxins-11-00520-s001.pdf]

Supplementary Materials: The Histone Deacetylases HosA and HdaA Affect the Phenotype and Transcriptomic and Metabolic Profiles of *Aspergillus niger*

Xuejie Li, Lijie Pan, Bin Wang and Li Pan

>A_niger_CBS_513_88 An07g08380
MARSAIVQEYAPPSSAPTFSVDRKVTHERQQNGISRPNGYRVSWHANPAVEPHHFGQSHPMKPWRLTLTKQLVMA
YGMHHAMDLYLARAATYEELADFHKSDYLDFLQQVVPGDMENPEQSENIARFNFGDDCPIFDGLYNYCSLYAGGT
IDAARKLCNNQSEIAVNWSGGLHHAKKAEASGFCYINDIVLGILQLLRLHPRVMYIDIDVHHGDGVEQAFWSTDR
VLTVSFHKYDKDNFFPGTGGLDSTGPTHPLNPGAHHSVNVPLHDGIDDESYIQLFRDVVGSCVEVYRPGAIVLQCG
ADSLGCDRLGCFNLNVAAHGACVAFVKTFNLPLLVVGGGGYTPRNVSRAWAHETSILIDAQDTIDPHIPETVTFRN
HFGPDFSLFPPLSEMRKLENKNPRTYLAGLLQSIREQLRYIQGAPSVQMSFIPPDILGLREDTEKEIEEETAMMEEEREE
RSGGGSLGKSSRRRDLERGAGQRGELF *
>A_niger_CBS_513_88 An07g07850
MSNSLAPLDPITNGPADRNKKVAYFYDSDVGNYAYVSGHPMKPHRIRMAHSLVMNYGLYKKMEIYRAKPASKYE
MTQFHTDEYIDFLSKVTPDNMDSFAKEQSKYNVGDDCPVFDGLFEFCGISAGGSMEGAARLNRNKCDIAVNWAGG
LHHAKKSEASGFCYVNDIVLGILELLRFKQRVLYVDIDVHHGDGVEEAFYTTDRVMTVSFHKYGEYFPGTGELRDIG
VGQGKHYAVNFPLRDGIDDVSYKSIFEPVIKSVMEWYRPEAVVLQCGGDSLSGDRLGCFNLSMRGHANCVNFVKSF
NLPTMILGGGGYTMRNVARTWAFETGILVGDTLGAELPYNDYYEYFAPDYELDVRPSNMDNANTKEYLDKIRTQV
VENLKRTAFAPSVQMTEVPREPLVEGMDDEADAILDDLDEDENKDKRFTKRRFDQYIEKPGELSDSDDEELAGANG
VRRQPNALKRRNQVNYRNLDVNDSGLESGMATPQDASSLPDDDMDTTADAKMTEAPEPETEAQATPSAADAPSR
ADEASAAEQTEMAVDGPETTAASAPISRQPSPKPQDEDTTMVDAGEVAPETEKSEAAPEGAAEEEEEKKKPSEETPA
ADKPATEETPATKDESPAKETTEAADTAEAETKPAEASEPSETKDKTPEAAKDESGEAKSKEPSAEAAGATEAEKTE
EASKTEE *
>A_niger_CBS_513_88 An16g01840
MAMDEDEDTVMGEAGSHHPTSVSSHQWPSNGPSSQGAGQLNGSSDPSIAPPLLGPTMKLPLSVHSGDPRPENTAS
AEDQGVSPPKSLPKSDSGDATTEDFDKQDSLVEDNSDWTDEDTVARTGLPIASLPSGLCYDVQMRYHCEVRPTADV
HPEDPRRIYYIYKELCRAGLVDDPESCRPLVSRPLKRINARNATEAEISLIHTPDHFAFVESTKDMSDDELIALEHTRDS
IYFNKLTFASSILSVGGAIETCLAVATRKVKNAIAVIRPPGHHAEHDKTMGFCLFNNVSVAARVCQNRLGDSCRKILI
LDWMGNSTLEGKKVTGITVVKALVSEADVFISNQCEPVASGFDAAAGDELGGCFVTPSCYAHMTHMLMTLANGK
VAVCLEGGYNFRSISKSALAVTKTLMGDPPDRLHTTSPSALATTTVRRVMMIQSHYWHCMYPKGPPQDGLFTDRLH
DVIRAYQSKQLYDNYKLTSLFIYRTAISRSFENQVLASSNYHQTVPLVVIFHDPPEIMGLPHPVTNKLEAHNCWLAD
VMKDYIGWIVSKGYAVIDVNIPKHVTIDPSSGKYEEEDENRPTATEELAGYLWDNYIEPSEATEIFFMGVGNAFYGV
ANLLINRETLYKRVNSVVSFVAENPVRAIASHTQVWLSRWYKDNSLVFVSHTHGVWNNVETRRKPSKRYGQLVQS
PKASLSEMLMHHKEEVYQWISERADVQDSEETEDEKKPKSKSRSPTKENQTEPRSVPG *
>A_niger_CBS_513_88 An14g00560
MDPSKRHEPTSFTAASSTPATGPPLSTDDATSSTHRLSHPSLHAMSLPPSSPGLSSAALLSPSDPPARAVATQAHPTPR
RIPSSSSLAEERRKSSPSLKKRSSTASLRSNRGASTSPRPSLSRHSSSSFGTSPTSASATHPNMAKKAHRTVSAEHPVTTA
ASIAAESFRKEVDWHQSAKLQSQTLVLVHDACYGHRFSRPRTSRAALNSIVERPERIQASVLGISAAYVRLARRHAG
GAFAPHPDLNPQQLPVPPFHIRKTARTLSLSSPAVTHVHGSKWMEDLKTMCDAAESRLALNGKELVRPRSAGKDS
AATNAPAFHEGDLYLCSESLSAFEGALGGVSEGVDAVMGPAPTKRAFVCIRPPGHHCSAGHPSGFCWINNVHVGIS
YAAMTHGLTHAAILDFDLHHGDGSQDIAWQQNHRAVTAPRNAAAHRKTMVGYFSLHDINSYPCEMGEPEKVRN
ASVCIDKAHGQSIWNVHLEPWKSDKEFWDLYAAKYTVLIDKARAFLRMHTERLANTPNGPPPKAAIFISAGFDASE
WEGAGMQRHQVNVPTEFYARFTADVVRMANEEGLGTDGRIISVLEGGYSNRALTSVICTGLGDAKGSTHDVDEQQ
INRLASEMTDRLGLSNSAEGRSVTEPVYNSEWWSPGVLEELEALVYSPAAPVKPREKAAPTYLTTTQSFSAKVVNPPR
DRRSTGSHSSPEPEAPSPPPPVSWATATHELCKVLIPTGRQTTSCQPEELNAEASRLRRERQTAVDAASTSATTAAVA
AAAAAAATATATAAEDSRMKLRVRKPKPSLPLSPSAETVKRPAARGSRRTTIDTAPDTAPAVQTPARVTRRKSAGA
GSAASTPEPTGPRPATSATVRKTNTSQSSTPRLPSSPNTPPVPRVPPAFLSAESEESPNPDGPQDDLDSLTAGVRKLHIK
LKVPSPEENAARERERERRIAEERKRTASKATSKTQTQTQTPKSPRKPAGRAQTSSASKTASRSAAKPAPVAIHPGTE
AKDEPSIVTPVTSSPPSVGKIESNTTNGWDRGESEAAAGMVMSPPLTPGRPPSALYSPPGMTLARDGLPVMMSSGVIP

FADTVRGMQEEEEQKGVNATHISEQSPTRSDKVVHRHAESFASPHDSRRRALPASARSIINDLSNLRRITPSYNLTLSF
PSPNSHSQSTIISRIPKCPQYPPPPPPPTKKHHQQQQQTPTSTPSARTARSQPATNSTSSRSAAPPARGHSVSSTAPKQ
RISAQTHVSRHHHPPPPPPPHLQPSSKNPPSTPQINAHTSHAKRSSIHSPTPACGAISAGRSIVSNIG *
>A_niger_CBS_513_88 An11g06980
MAPPAIPAADLSSFTTYLKGCRRVIALLGAGISASSGLPTFRGAGGLWRSYDATDLATPEAFDANPDLVWQFYSYRR
HMALKAQPNRAHYALAELARKNKDFITLSQNVDDRTGEEASRSIASALGNNEDEADVSDERVPLSAVPYEELPHCP
ECKDGLLRPGVVWFGESLPSHTIDYVDEWLNKGKVDLILVVGTSSRVYPAAGYVDKARSKGARVAVVNMDRNDV
GSSGLKPGDWFFEGDAGTIVPEILKGVIGDI *
>A_niger_CBS_513_88 An08g02500
MAPRRTATTSSTTTTTTTTTSTTHSTTNSSRSSPKSLLASLGMDDDSDCSSLSSAPASPLAPPGFYPSPPPSQDADEPSA
ARSQDDLPPARKKRRVAAPKERRTQHLDLSATAGLSYTEQQSQIDYLVKTLQRHRKIVVIAGAGISTSAGIPDFRSTD
GLFKSLQKKHNLKASGKLLFDAAVYQDEALTASFQDMVRSLSEEAAKTSPTAFHHMLARLGQENRLTRLYTQNID
GIETSMPPLATQIPLNVKAPWPRTIQLHGSLDKMVCQKCRHLSDFDAGLFDRPDAPECPECCRNNQFRMETGQRSH
GIGKMRPRIVLYNEHNPDEEAITSVMNADIRSRPDALIVVGTSLKIPGVRRLVKSLCSVIRSRRNGVTMWINNEYPTG
KEFEDCWDLMVKGDCERSCTTGQPQTVGCAGRQGEVSVVVTPSKKRPTPTGFLTPSSSYEDESKPLAKKGRPNPAS
NGRSITDVLQAAKTDGPKKAAPKKAAPKKAAPKGRSKKVEPAKNSKINNFGRVTKAQKAATSDKSTKLDKDDTK
AMHPLAPGAARNNGPNQTFPKLVGKGASTPPPSGRWRQNETISPDSIPRGMNLLLNEPTA *
>A_niger_CBS_513_88 An01g07390
MDLVSAPSGEDSSLKDPVVQVEAVDMASAVSKAGTATENTAAAKEEVTQSEYETDSDGSGDEWETQSLYEDAIQVL
RDDQLRDGVPDACTLEEAVAYRKRLHEVGKAAFVEETLGQDKVTARKLCTAFGILPPAFLADAPDEAFHPLLAIAIS
REFSRRPKLPQYNTIDDAVKLLKESKNIVVLTGAGISTSLGIPDFRSKDTGLYSQLAHLGLSDPQEVFDIQVFREDPSIFF
SIAKDILPTEKKFSPTHAFIRVLQDKGKLLTNYTQNIDNIEANAGVLPENIVQCHGSFATATCVKCQYKVKGDEIFDEI
KKGVIPQCDSCRKRIAEDSGIKRKRSSNGVHKNRKDNDGDSTDDDYEIPTPGVMKPDITFFGEDLPDEFGRRLLHHD
RDQVDLVIVIGTSLKVAPVAEVPGVLPRTIPQLYISRTPVSHTEFDIDLLGDCDVVVSELCRRAGWELKHEMIPEDEKV
EVLPVEVSVGHLVGRHSMYEGWLMGA *
>A_niger_CBS_513_88 An09g05710
MSRKRKASSTIYTSHGSHKRKKRWRYQHVVHSSDTETDNDSEALGADEWLINCILDESESQYLIDWEGPWTPTWEP
KENANDVAVQVWEDKKRRDQVQEEFVRCNTPLPLPPTPPPGSSVQERCRDSLGPNFSQPQTEAPTDEPPPRQSPTSP
PFVLPSTEPEPSLCLDDDARGILRSSSVNTEVPSHPPQSDIDNLLNPEVLPTARATEVPAIFSTPREPRVSDEVEETPTTP
CRAGNTGTPNVAEGIPETPHNESSAAPANIPELLPANPAEGADEIPETPPGLVDEPQLFRRASTVPESIAVNSTHTPA
ARISPAPSFRSLVDQLISSISRPSMDTQEKEGPSLSETMEKYSQFEGSTPREKLRNAYAQLRAKANPGPESSATPSSAGDI
EPSAPVSIPETTAPLSVRVDKEPTHPKDNEANQDTTIPPVEPEEIGPPQTIQPSALSMHFAEEPTPGSVHLGPSEFAVPL
PMDSRVKDDYERVLTSESQSIRDFLGVTSSQPDTTEYQRDSLLPKMHDILDRLSNISTHPDLNIAEHINSESDLGREAA
WAEYSSAKFLLLSYLLETASMRDIHLVIMVQGEKTQRVVERYLMGKGLLYTRPRQEMGSGTNIEVSMVKDSLSFGIQ
TTRNDGVMETYKPPAAIIALDKSFSSKTPTVEHMRTTFARNGNLLPVVRLLVSNSSEHIELCFPGLSEPERLRTVLQYSI
RLRDVVGDLQDDALGVQEDAEELLSCLVSDNFNAHWSLPHIEPLHILSPEQLESSQQELQSRPGVDIQLTNPQAQKR
AFAEDEVEQSAKRMKTEEPTQEPTQSTESTGFPSQPLNGGLQTLENHLVQMQNSHAAELEKLQKALAGAQARLQE
RENVLEKLQHRYETRTKDFHKLRREHDRLTQAKTTSDQRVEKQKEELMKLKDERTQLRHELEEARASLKAGGGSA
AELESAREDIRRLTKDNASLERKAEYEARQAEYTREQYQTASTVAAQSGNELRQLRSENETLKRKVEGDATHLRELN
AKNDEARHLARVLELEATLTSREDLLRRKEEELREIRKNRPSTRSTSTQPRSPKWTAGNSRPTSPGINNGSSLAGRGSA
LRFSSEMPL *

Figure S1. Amino acid sequences of eight HDACs in *A. niger* FGSC A1279 obtained from ASPGD. * represents termination codon.

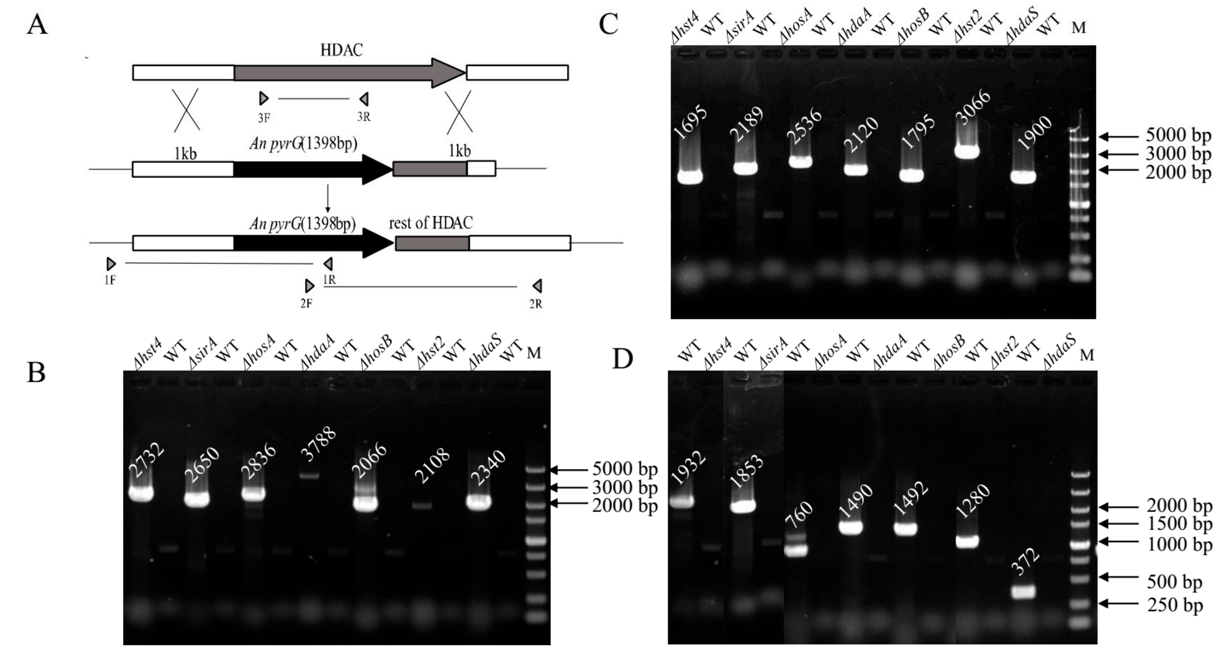

Figure S2. Disruption of HDAC genes in *A. niger* FGSC A1279. (**A**) Each HDAC coding sequence was replaced with the pyrG selection marker by double homologous recombination. Positive deletion strains were confirmed by PCR screening with three different primer pairs. Small triangles indicate the positions of the PCR primers that were used to identify the HDAC disruption strain. Straight lines indicate the amplicon lengths for PCRs using the corresponding primers. (**B**, **C**, **D**) Agarose gel electrophoresis of the PCR products using primers 1F and 1R, 2F and 2R, and 3F and 3R respectively, which was used to confirm that the histone deacetylases disruption mutant is homokaryotic. The template of each PCR was marked on each lane. Lane M, DL5000 DNA Marker (TaKaRa).

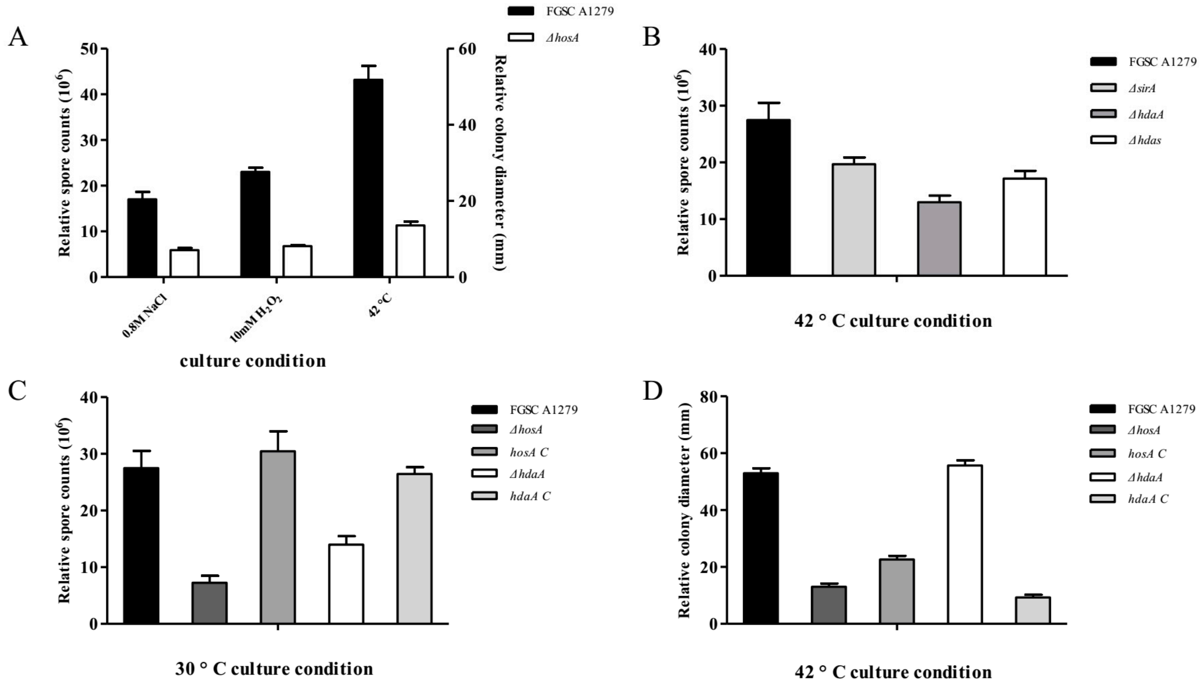

Figure S3. Statistical analysis of spore count and colony diameter. (**A**) Quantification of rate of conidiation under condition of 0.8M NaCl or 10mM H_2O_2 and colony diameter at 42 °C of FGSC A1279 and *ΔhosA* strains. (**B**) Graphs represent the number of conidia at 42 °C. (**C**) Graphs represent the number of conidia at 30 °C. *hosA C*: *hosA* complementation strain, *hdaA C*: *hdaA* complementation strain. (**D**) Graphs represent radial growth at 42 °C. All data are represented as means ± s.d. (*n* = 3).

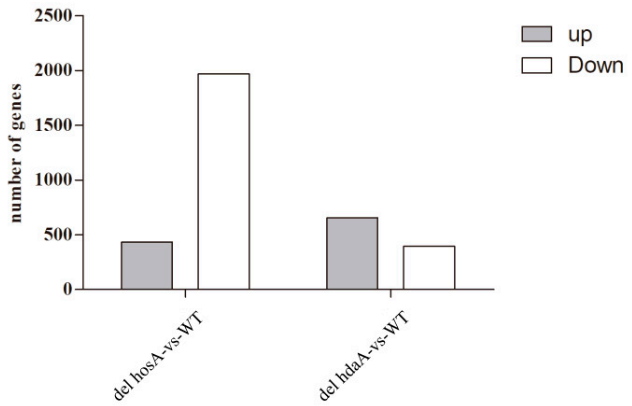

Figure S4. Overview of the number of differentially expressed genes via pairwise comparisons of the *ΔhosA* mutant and wild strains, *ΔhdaA* mutant and wild strains.

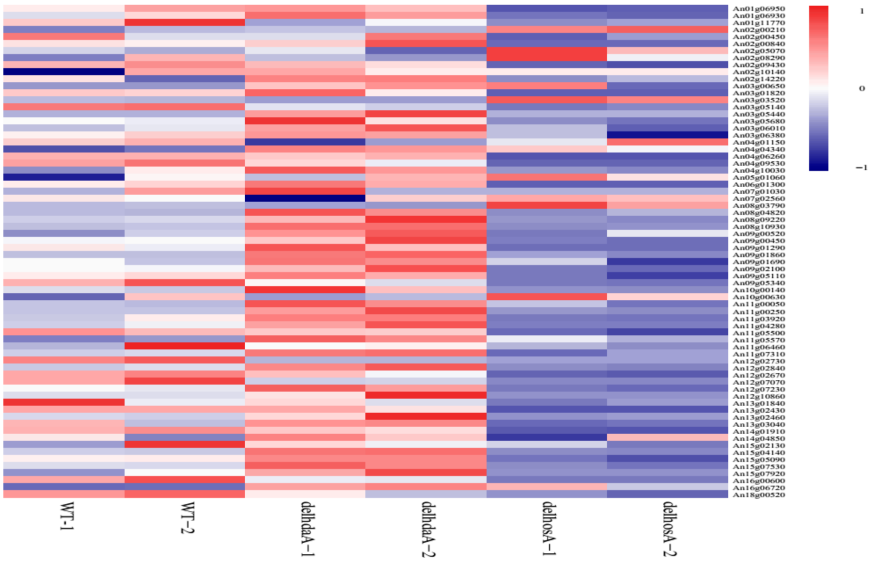

Figure S5. Heatmaps display expression of backbone genes of SMs biosynthesis gene clusters in wild strains, *ΔhosA* and *ΔhdaA* mutants after seven days of growth on WATM at 30°C.

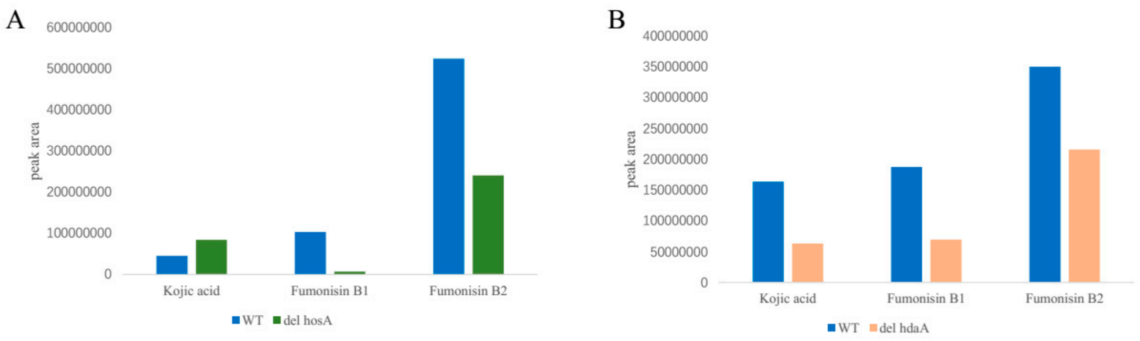

Figure S6. Analysis of the expression levels of kojic acid (KA), fumonisin B1 and fumonisin B2 in the wild-type strains and the *ΔhosA* and *ΔhdaA* mutants. (**A**) Bar graphs show the expression values (peak area) for KA, fumonisin B1 and fumonisin B2 in the wild-type strains and *ΔhosA* mutants after seven days of growth in WATM at 30 °C. (**B**) Bar graphs show the expression values (peak area) for KA, fumonisin B1 and fumonisin B2 in wild-type strains and *ΔhdaA* mutants after seven days of growth in WATM at 30 °C (n = 1).

Table S1. Summary of sequencing and reads mapping.

Samples	Total Clean Reads (M)	Total Mapping (%)	Uniquely Mapping (%)
WT-1	23.1	91.22	88.19
WT-2	23.09	91.18	88.14
ΔhdaA-1	22.91	90.81	87.65
ΔhdaA -2	22.99	90.82	87.72
ΔhosA-1	23.06	91.56	88.5
ΔhosA-2	23.1	91.56	88.5

Table S2. Expression level of genes related to asexual or sexual reproduction.

Proteins	Genes ID	FPKM					
		WT-1	WT-2	del hosA-1	del hosA-2	del hdaA-1	del hdaA-2
brlA	An01g10540	19.05	23.36	0.3	0.45	6.43	4.76
abaA /tec1	An01g03750	4.77	4.59	2.19	2.12	4.15	4.35
dewA	An03g02400	462.53	560.82	1.04	0.69	48.54	28.14
yA	An01g13660	149.54	172.28	0.07	0.35	99.89	77.81
wA	An12g02050	0.03	0.07	0.02	0.02	0.29	0.24
ppoC	An02g07930	31.76	38.29	4.21	3.29	22.78	25.29
ppoD	An12g01320	49.96	57.96	2.57	1.48	30.87	32.78
nsdD	An02g09610	76.97	76.28	24.49	34.82	54.69	65.15
Nc asd-1	An14g01130	1.27	0.95	0.32	0.39	0.72	1.04

Table S3. Expression level of genes related to cell wall biosynthesis.

Proteins	Genes ID	FPKM					
		WT-1	WT-2	delhdaA-1	delhdaA-2	delhosA-1	delhosA-2
GelB	An08g07350	134.64	140.92	89.91	92.14	64.81	66.14
BgtA	An08g03580	260.41	261.51	145.87	110.56	4.72	4.54
CrhD	An01g11010	398.43	295.43	410.83	518.3	167.63	161.94
ExgA	An18g04100	18.86	23.11	8.37	6.98	1.28	1.93
BxgA	An01g12450	115.61	123.43	105.44	125.79	56.47	51.41
BgxB	An02g13180	24.64	29.61	14.46	11.64	15.42	11.96
AgsD	An02g03260	27.16	38.63	24.32	20.83	0.09	0.09
AgsC	An12g02450	9.05	11.64	12.91	16.77	5.13	2.9
AgsB	An15g07810	17.71	23.79	28.91	28.54	4.97	3.49
agtB	An12g02460	76.01	84.82	125.27	131.69	25.47	16.67
agtC	An15g07800	33.98	36.05	45.49	42.92	11.45	10.1
AgnE	An15g04760	124.53	129.22	140.66	103.68	0	0.08
ChsG	An08g05290	7.77	11.08	6.65	6.3	3.57	3.07
CtcA	An09g06400	47.45	20.77	7.15	8.77	20.82	33.79
CtcB	An09g05920	14.91	15.09	7.03	4.95	4.05	3.9
CfcI	An02g13580	13.31	14.24	3.57	3.87	0.59	0.38
KslA	An02g03980	18.47	19.93	11.56	13.87	7.39	8.91
DfgA	An18g01410	0.56	1.36	0.14	0	0.05	0
DfgB	An03g00740	5.7	6.26	3.67	3.34	1.61	1.51
DfgF	An06g00360	17.31	20.06	33.04	35.47	5.48	8.21
hypB	An01g10940	28.65	30.76	1.87	0.94	2.33	3.01
hypC	An03g02360	350.38	417.19	44.61	20.6	0	0
hypD	An03g02400	462.53	560.82	48.54	28.14	1.04	0.69
hypF	An07g03340	195.05	199.11	47.8	44.43	31.46	32.18
hypH	An09g05530	4.99	6.72	2.07	1.43	0.54	1.19

Table S4. Expression level of backbone genes in SMs gene clusters.

Backbone Genes	Cluster Type	FPKM					
		WT-1	WT-2	del hosA-1	del hosA-2	delhdaA-1	delhdaA-2
An01g06950	PKS	0.27	0.37	0.07	0.08	0.38	0.33
An01g06930	PKS	400.35	548.66	198.70	160.55	794.12	693.34
An01g11770	NRPS-Like	0.17	0.34	0.00	0.02	0.02	0.10
An02g00210	NRPS-Like	116.97	132.17	189.52	199.39	134.87	130.26
An02g00450	PKS	0.05	0.02	0.00	0.01	0.02	0.05
An02g00840	NRPS-Like	52.83	47.80	1.96	1.95	58.78	99.72
An02g05070	NRPS	0.61	0.54	1.07	0.83	0.65	0.42
An02g08290	PKS-NRPS hybrid	10.13	16.27	20.13	13.36	11.93	10.77
An02g09430	PKS	3.49	4.02	0.85	0.62	3.45	2.97
An02g10140	NRPS-Like	0.00	0.09	0.07	0.07	0.09	0.07
An02g14220	PKS-Like	10.52	8.59	9.05	9.47	11.71	11.81
An03g00650	NRPS	49.36	49.53	77.29	44.10	68.42	74.17
An03g01820	PKS	2.41	2.30	0.00	0.00	3.69	1.98
An03g03520	NRPS	52.12	51.23	85.31	78.90	48.85	49.01
An03g05140	PKS	2.46	2.53	0.43	0.55	1.17	1.01
An03g05440	PKS	0.00	0.00	0.00	0.00	0.04	0.06

An03g05680	NRPS-Like	0.25	0.23	0.06	0.02	0.67	0.32
An03g06010	NRPS	0.02	0.03	0.02	0.00	0.06	0.08
An03g06380	PKS	15.55	16.65	13.54	9.22	18.36	18.07
An04g01150	NRPS-Like	0.43	0.47	0.35	0.55	0.17	0.27
An04g04340	PKS	2.67	3.06	5.03	4.32	5.82	5.61
An04g06260	NRPS	0.09	0.09	0.01	0.01	0.08	0.09
An04g09530	PKS	1.44	1.72	0.00	0.00	1.08	0.74
An04g10030	PKS	0.40	0.75	0.43	0.38	1.23	1.05
An05g01060	NRPS	1.02	1.99	2.65	2.09	1.67	2.31
An06g01300	NRPS	22.07	24.49	7.82	7.72	33.20	28.26
An07g01030	PKS	0.00	0.02	0.00	0.00	0.03	0.00
An07g02560	DMAT *	2.87	2.72	3.27	3.14	1.29	3.00
An08g03790	PKS	10.38	11.32	44.38	31.74	8.16	7.34
An08g04820	NRPS-Like	0.62	0.60	0.51	0.60	1.29	1.12
An08g09220	NRPS-Like	3.25	3.50	1.83	1.41	6.74	10.78
An08g10930	PKS-Like	0.88	0.93	0.22	0.31	4.09	4.12
An09g00520	NRPS	0.05	0.08	0.04	0.09	0.16	0.19
An09g00450	NRPS-Like	7.17	7.52	3.28	2.75	9.62	14.54
An09g01290	PKS	0.07	0.05	0.00	0.00	0.14	0.09
An09g01860	PKS	0.02	0.02	0.01	0.00	0.11	0.12
An09g01690	NRPS	1.29	1.13	1.17	0.75	1.74	1.66
An09g02100	PKS-Like	0.15	0.14	0.06	0.05	0.20	0.28
An09g05110	NRPS-Like	1.21	1.28	0.69	0.50	1.63	1.44
An09g05340	PKS	0.74	1.08	0.04	0.03	0.48	0.35
An10g00140	PKS	3.14	2.26	0.02	0.00	14.01	7.55
An10g00630	PKS-Like	0.56	1.14	1.47	1.08	0.71	0.77
An11g00050	NRPS	0.13	0.12	0.13	0.07	0.32	0.27
An11g00250	PKS-NRPS hybrid	0.62	0.62	0.30	0.22	1.99	2.68
An11g03920	PKS	0.11	0.20	0.02	0.01	0.37	0.37
An11g04280	PKS	0.34	0.45	0.14	0.14	0.78	1.02
An11g05500	NRPS-Like	5.87	5.47	3.54	3.23	5.33	5.25
An11g05570	PKS	0.92	0.97	1.13	1.01	1.51	1.42
An11g06460	PKS-NRPS hybrid	0.50	1.72	0.50	0.38	0.80	0.83
An11g07310	PKS	0.08	0.09	0.01	0.05	0.23	0.24
An12g02730	PKS	2.21	2.68	0.00	0.00	0.13	0.14
An12g02840	NRPS	1.01	1.22	0.35	0.23	3.14	3.73
An12g02670	PKS	0.75	0.87	0.04	0.01	0.68	0.45
An12g07070	PKS	1.32	1.99	0.00	0.00	0.41	0.44
An12g07230	NRPS	1.94	1.72	0.64	0.53	3.70	3.09
An12g10860	NRPS-Like	0.02	0.02	0.00	0.00	0.04	0.10
An13g01840	DMAT *	5.88	2.37	0.78	1.31	3.25	2.17
An13g02430	PKS	6.03	5.97	0.55	0.54	5.96	4.63
An13g02460	NRPS-Like	0.12	0.10	0.00	0.02	0.28	0.67
An13g03040	NRPS	1.78	0.69	0.09	0.19	2.07	2.16
An14g01910	PKS	9.74	11.28	0.47	0.30	8.83	7.77
An14g04850	PKS	1.99	1.78	1.68	2.06	2.15	2.04
An15g02130	PKS	4.25	11.40	5.38	3.45	7.26	6.02
An15g04140	PKS	13.62	13.10	5.98	5.05	40.11	40.73
An15g05090	PKS	1.60	1.64	0.45	0.18	2.51	2.53

An15g07530	NRPS	1.25	1.22	0.91	0.79	2.21	2.02
An15g07920	PKS	0.02	0.03	0.02	0.02	0.04	0.05
An16g00600	NRPS-Like	33.66	47.51	2.82	2.64	17.76	16.72
An16g06720	NRPS	0.59	0.60	4.94	2.33	5.46	6.21
An18g00520	PKS-NRPS hybrid	0.19	0.23	0.03	0.00	0.12	0.06

*: Dimethylallyl tryptophan synthase.

Table S5. Data of metabolomic studies. Expression of kojic acid, fumonisin B1 and fumonisin B2 in wild strains and *ΔhosA* mutant.

Names	Formula	Molecular Weight	RT [min]	Pos WT	Pos del hosA
Kojic acid	C6 H6 O4	142.02646	4.411	45124755.87	83986818.87
Fumonisin B1	C34 H59 N O15	721.38558	11.678	103269356.1	6773931.209
Fumonisin B2	C34 H59 N O14	705.39025	11.819	524825567.1	240944030.1

Table S6. Data of LC-MS. Relative output of kojic acid, fumonisin B1 and fumonisin B2 in *ΔhosA* mutant/WT and *hosA C*/WT.

Name	Formula	Molecular Weight	RT [min]	Ratio: (*ΔhosA*)/(WT)	Ratio: (*hosA C*)/(WT)
Fumonisin B1	C34 H59 N O15	721.38687	12.23	0.01	0.583
Fumonisin B2	C34 H59 N O14	705.39218	12.343	0.032	0.685
Kojic acid	C6 H6 O4	142.02677	4.996	1.951	1.287

Table S7. Data of metabolomic studies. Expression of kojic acid, fumonisin B1 and fumonisin B2 in wild strains and *ΔhdaA* mutant.

Names	Formula	Molecular Weight	RT [min]	pos_WT	pos_del_hdaA
Kojic acid	C6 H6 O4	142.02664	5.033	163530429.9	63109327.37
Fumonisin B1	C34 H59 N O15	721.38623	11.71	187371433.7	69481438.94
Fumonisin B2	C34 H59 N O14	705.39122	11.776	349702627.4	215526568.1

Table S8. Data of LC-MS. Expression of kojic acid, fumonisin B1 and fumonisin B2 in in *ΔhdaA* mutant/WT and *hdaA C*/WT.

Name	Formula	Molecular Weight	RT [min]	Ratio: (*ΔhdaA*)/(WT)	Ratio: (*hdaA C*)/(WT)
Fumonisin B1	C34 H59 N O15	721.38686	12.248	0.146	0.257
Fumonisin B2	C34 H59 N O14	705.39218	12.371	0.22	0.41
Kojic acid	C6 H6 O4	142.02675	4.977	1.15	1.677
